# A *somewhat incredible interpretation* of ‘that’-clauses: Russell, non-existent kings and false objectives

**DOI:** 10.1007/s11229-026-05688-6

**Published:** 2026-07-02

**Authors:** Giulia Felappi

**Affiliations:** Department of Philosophy, University of Southampton, Avenue Campus, Highfield Rd, Southampton, SO17 1BF UK

**Keywords:** Russell, *On Denoting*, Propositions, ‘That’-clauses, Facts, False objectives

## Abstract

‘The present King of France’, Russell maintained in *On Denoting*, seems to force us into either of the following positions. Either we follow Meinong but then infringe the law of contradiction, or posit senses beyond references, but we seem never able to genuinely talk about them. Hence, Russell urged, it is worth considering ‘a somewhat incredible interpretation’ of definite descriptions. This is his famous theory of descriptions, now not considered to be incredible anymore. In this paper we will investigate whether and, if so, how some of *On Denoting*’s core claims can be applied not to the present King of France, but to propositions. We will see that following Russell on his considerations concerning the present King of France but not on parallel considerations concerning propositions and phrases that seem to refer to propositions is an unstable position, which Russell himself in *On Denoting*, and most of us today, seem to occupy. We will then discuss how to reach a more stable position. According to the account we will end up presenting, while there is some thing we believe when we believe truly, there is no thing we believe when we believe falsely. The account might look like a non-starter and goes against what Russell himself claimed. But, we will argue, we can find in *On Denoting* and in its theory of descriptions the resources to render such an account at least a bit less incredible.

## Introduction

‘The present King of France’, Russell maintained in *On Denoting*, seems to force us into either of the following positions. The first option seems to be to follow Meinong, and then have objects that are ‘apt to infringe the law of contradiction’ (1905, p. 483), since, Russell maintains, on a Meinongian take, the present King of France exists, and also does not exist. Alternatively, we can try, with Frege and pre-*On Denoting* Russell himself, to posit senses beyond references, but ‘the relation of the meaning to the denotation involves certain rather curious difficulties’ (ibid., p. 485) as we seem never able to genuinely talk about them. Neither option is palatable. Infringing the law is ‘intolerable’ (ibid., p. 483), and the difficulties senses give raise to are for Russell ‘sufficient to prove that the theory which leads to such difficulties must be wrong’ (ibid., p. 485). Hence, Russell urged, it is worth considering ‘a somewhat incredible interpretation’ (ibid., p. 482) of definite descriptions. This is his famous theory of descriptions. The theory has been criticised in a variety of ways then and now. Similarly, Russell’s claims concerning both apparent horns of the dilemma leading to his theory have been called into question, then and still now. Still, as stated on the ‘Descriptions’ entry of the *Stanford Encyclopedia of Philosophy*, the received view today is that

Russell’s core insight remains intact: The critical question is whether the sentences in which they appear are quantificational or referential, and Russell may well be right about the critical cases here. That is, many apparently referential constructions may in fact be quantificational (2023),

and Russell’s then somewhat incredible interpretation is now not considered to be incredible anymore.

In this paper we will investigate whether and, if so, how some of *On Denoting*’s core claims can be applied not to the present King of France, but to objects of another kind, that is, propositions. We will see that following Russell on the King of France but not on propositions is an unstable position (Sect. 2). We will then consider whether we can extract from Russell a way to reach a more stable position. While in *On Denoting* he was happy to admit propositions, after it, he himself eliminated propositions, in two radically different ways. But both these ways are usually considered unsatisfactory, so many would today argue that we cannot *straightforwardly* extract from Russell a way to reach a stable position (Sect. 3). Hence, in the rest of the paper, we will then consider whether one could extract from Russell a way to reach a more stable position in a *less straightforward* way. The intent is not historical. Rather we will consider whether we could learn from *On Denoting* how to reach a position more stable than the one many of us seem to occupy today. We will then discuss Russell’s *intact core insight* in relation to propositions and ‘that’-clauses, which do seem to refer to propositions. We will see that the accounts in the literature that take ‘that’-clauses as quantificational do not seem to be able, on their own, to address our issue (Sect. 4). We will then move to another point made in *On Denoting* and see that, by exploiting it when it comes to propositions and ‘that’-clauses, we can indeed reach an account leading to a more stable position. According to such an account, while there is some thing we believe when we believe something true, there is no thing we believe when we believe falsities. The account might look like a non-starter and goes against what Russell himself claimed. But, we will argue, we can find in *On Denoting* and in its theory of descriptions, now considered not incredible anymore, the resources to render such an account of propositions and ‘that’-clauses a bit less incredible (Sect. 5).

## From the present King of France to propositions

In *On Denoting*, Russell himself relied on propositions. Yet, as we will now see, they and the phrases we use which seem to refer to them appear to be subject to exactly the problems that led him to put forward his somewhat incredible interpretation of definite descriptions. In the case of descriptions, the two options seemed to Russell to either say that the denoted object somehow exists, but then contradictions would ensue, or to introduce senses beyond references, but those lead to difficulties. In order for these two options to be independent from each other when it comes to propositions and the phrases that seem to refer to them, they would then need to be to posit propositions not made of senses, or to introduce senses beyond references.

Let’s start from the second option. This is also an option for propositions, of course. But if senses are to be avoided because they lead to *rather curious difficulties*, they are to be avoided both when it comes to definite descriptions and when it comes to ‘that’-clauses.

Let’s then move to the first option. A clear way to characterize propositions in such a way that they are not made of senses is to go with what nowadays goes under the label of *Russellian propositions*, made of things like you, me, mountains and snow. Now, these propositions, as shown by Schiffer (1987, p. 463), do seem to infringe the law of contradiction. The contradiction is not as straightforward as the Russellian proposition existing and also not existing, but there seem to be contradictions nonetheless. For you know that Cicero is Tully. Now suppose that Berty believes that Cicero is not Tully. If we go with Russellian propositions, we should conclude that Berty believes that Cicero is not Cicero. Yet, Berty keeps telling us that he is unwilling to claim that Cicero is not Cicero. Berty is sincere and not reticent. Given Kripke’s strong disquotational principle – *A normal English speaker who is not reticent will be disposed to sincere reflective assent to ‘p’ if and only if he believes that p* (1979/2012, p. 138) – it seems that we should also conclude that Berty does *not* believe that Cicero is not Cicero. So it seems that the proposition that Cicero is not Cicero is both *believed* and *not believed* by Berty. We just infringed the law of contradiction. The contradiction can be avoided, of course, in a number of ways. One for all, with Burge (1982, p. 288), we can simply reject the strong disquotational principle. But the apparent contradiction created by Meinongian kings of France can be avoided too. What Russell claimed is that Meinong’s view is committed to claiming ‘that the existent present King of France exists, and also does not exist; that the round square is round, and also not round’ (1905, p. 483). But this is far from obvious. Adjusting what he urged concerning Meinongian objectives to fit Meinongian kings, he urged that he could not see how a non-existent object ‘differs from no object at all’ (1904, p. 509). But Meinongians could and can indeed see the difference. Exactly as there is a gap in the train of thought to the conclusion that propositions not made of senses infringe the law, there is a gap in Russell’s own train of thought to the conclusion that Meinongian kings of France infringe the law. For Russell this is the chief objection to Meinongian kings, but it seems hardly able to rule them out.

Russell also adds that Meinong’s view is ‘in itself a difficult view’ (ibid.). He does not say why he thinks so, but one can imagine that, at play here, there is his *vivid sense of reality*, which he will invoke years later. Now, in the case of descriptions, the problem is of course created by descriptions denoting non-existent objects – nobody would go into an incredible interpretation for ‘The present King of the United Kingdom’. The equivalent in the realm of propositions is false propositions. Indeed, as Dorothy Wrinch urged while discussing later Russellian takes on judgment, ‘the difficult case is the case in which the judgment is false and then there is no fact. It would be a matter of little difficulty to get out a large class of theories of judgment, if judgments were all true’ (1919, p. 324). For our purposes, the question to ask is the following: Are false propositions, that is, false Meinongian objectives, so different in kind from Meinongian kings that one can easily be happy to admit the former but not the latter? False propositions, whether made of things like you and me, or senses, seem indeed ‘almost incredible’ (1910, p. 152)^1^, as Russell would tell us explicitly after *On Denoting*, almost exactly as difficult to accept as Meinongian kings and the alleged denotations of any non-denoting definite description. Concerning the claim that a false belief is a relation to an object, Russell claimed in 1912 that it ‘is almost as difficult to suppose that there is such an object as this … as it was to suppose that there is ‘Desdemona’s love for Cassio’’. (1912, p. 124). In 1918, Russell seems to even drop the ‘almost’ in the quote above:

it does not seem to me very plausible to say that in addition to facts there are also these curious shadowy things going about such as ‘That today is Wednesday’ when in fact it is Tuesday. I cannot believe they go about the real world. It is more than one can manage to believe, and I do think no person with a vivid sense of reality can imagine it. One of the difficulties of the study of logic is that it is an exceedingly abstract study dealing with the most abstract things imaginable, and yet you cannot pursue it properly unless you have a vivid instinct as to what is real. … when I say ‘Desdemona loves Cassio’, it seems as if you have a non-existent love between Desdemona and Cassio, but that is just as wrong as a non-existent unicorn. (1918a, pp. 55–66)

There might well be other trains of thought leading to Russell’s theory of descriptions that do not easily extend from the present King of France to propositions. Still, a dislike for the present King of France surely played a crucial role in turning Russell’s somehow incredible interpretation of descriptions into something not incredible anymore. But if we dislike the present King of France, it is unclear why we should so easily accept false propositions.

One might at this point urge, with Johnston and Sullivan, that ‘[t]he parallel between true and false judgments requires that the judgment that Charles I died in his bed also has a proposition as its objective, but it clearly does not require that this proposition be describable as a fact’. (2018, p. 158) Said differently, if true propositions were not to be equated to facts, then it is unclear what the problem with false propositions would be. For example, it is unclear why propositions taken to be abstract objects, made of things like you and me, properties and relations, taken in an ordered manner, another view that goes under the label of ‘Russellian’, would be problematic. On further consideration, though, it seems that there is still room to think that false objectives are as problematic and shadowy kinds, and exactly because some of the falsities we seem to believe are apparently about shadowy entities. Remember that here we cannot avail ourselves of senses – we are considering the first option, not the option of relying on senses, and moreover if we should avoid senses for definite descriptions, they are to be avoided everywhere. But then take something like ‘that Santa Claus is coming’. Russell in *On Denoting* claimed that ‘a proposition about Apollo means what we get by substituting what the classical dictionary tells us is meant by Apollo, say ‘the sun god’’ (1905, p. 491), and most probably he would have been happy to claim something similar for ‘Santa Claus’. But in the position in which we currently are, the widespread view is instead that this is not always the case: ‘certain singular terms refer directly … the proposition expressed by a sentence containing such a term would involve individuals directly’ (Kaplan, 1977, p. 483). If we combine this view on names, currently standard, at least for some of them, and the claim the propositions are abstract objects of the Russellian kind just indicated, then some ‘that’-clauses, involving directly referential empty names, should be taken to and are usually taken to express *gappy propositions*, or *unfilled propositions*, as those names do not contribute anything to the propositions expressed. Now, at least at first sight, it does not seem that our vivid sense of reality can be at peace with gappy propositions. It can maybe cope well with an abstract object made up of the empty set and something else. But this would not be a gappy proposition. For the theory of direct reference for empty names does not claim that a directly referential empty name refers to the empty set, but that it refers to no thing, so that gappy propositions are structured entities parts of which are literally gaps (Braun, 1993, p. 463). Maybe we can make sense of the gap, of the nothing an empty name refers to, as something able to be a part of an abstract object, but these gaps surely do not seem at first any better than a non-existent unicorn. Moreover, if our sense of reality can tolerate them, it is unclear why it would not be able to tolerate non-existent unicorns as well. Hence, it does not seem that keeping propositions apart from facts surely makes us reach a more stable position, as, at least prima facie, our vivid sense of reality seems offended again. Moreover, the infringement of the law of contradiction we obtained out of Schiffer’s considerations does not depend on whether we take propositions to be facts, or something else. Again, the contradictions can be avoided, but so can the ones seemingly created by shadowy kings of France.

## Russell’s rejections of propositions

In *On Denoting*, Russell himself did not take false propositions to offend his vivid sense of reality, as he rather kept having them. But after *On Denoting*, he then tentatively moved to the multiple relation theory of judgment (1907, 1910, 1912, 1913, 1917, 1918a; Whitehead & Russell, 1910), where propositions as objects of thoughts are dissolved, in favour of taking subjects to be related to a multiplicity of objects like you, me, mountains, snows, properties and relations. It is far from clear why Russell moved to the multiple relation theory of judgment (Alford-Duguid & Amijee, 2022; Proops, 2011)^2^ and it is not to be assumed that he did so because of the considerations we saw in Sect. 2. We will not go into this here. Be it as it may, Russell’s multiple relation theory of judgment surely eliminates propositions and Meinongian objectives in a way similar to how one could eliminate Meinongian kings. But it also surely did not receive the acclamation Russell’s theory of descriptions did and still does receive. Ramsey maintained that Russell merely stated that ‘a judgment has not one object but many, to which the mental factor is multiple related’ and further claimed that

to leave it at that, as he did, cannot be regarded as satisfactory. … Similarly, a theory of descriptions which contented itself with observing that ‘the King of France is wise’ could be regarded as asserting a possibly complex multiple relation between kingship, France and wisdom, would be miserably inferior to Mr. Russell’s theory, which explains exactly what relation it is. (Ramsey, 1927, p. 157).

Wittgenstein raised a cluster of objections to the view, which famously appear to have ‘paralysed’ Russell, and even made him feel ‘ready for suicide’.^3^ Whether it was because of these objections or not, Russell himself ended up rejecting the view. Forgetting about Russell’s official and unofficial reasons for his dissatisfaction with the view, it is certainly the case today that the view is widely considered ‘impossible’, as Russell himself ended up claiming (1919, p. 27). The predominant attitude toward the multiple relation theory is nicely summarised in Armstrong’s take on the matter: ‘Despite its ingenuity, Russell’s solution seems unworkable. … it would be wasted labour to go over the ground again’ (1973, p. 44). The multiple relation theory is indeed considered by most as a truly incredible interpretation.

Russell did not try to eliminate propositions in only one way. Very briefly, he arguably also played with the idea that there is one thing we believe when we believe that *p*, but that thing is not a proposition made of either senses or things like you, me, mountains, snow, properties and relations, but rather a linguistic item, a sentence made up of words (see Bostock, 2012, ch. 12 and Shieh, 2024, pp. 84–87 for discussion). His motivation was not connected to *On Denoting*’s considerations, but rather to his investigation into behaviourism, but this does not matter for our purposes. What matters is that in a manuscript from 1918, while dealing with what he considers to be what is ‘most stubbornly ‘mental’’ (1918b, p. 271), that is, what ‘comes under the head of ‘propositional occurrences’, i.e. occurrences which one would naturally express in such forms as ‘*A* believes that so-and-so’ or ‘*A* desires that so-and-so’ and so on’ (ibid.), he in fact put forward the following three claims:

We can consider a proposition or desire it or … believe a proposition (ibid.)

a proposition is a series of words (ibid.)^4^

A single word is a class of similar noises. (ibid., p. 270)

Hence, arguably, Russell briefly considered taking the objects of the attitudes to be linguistic items, sentences made of words, which themselves would be classes of noises (and, possibly, marks). Wrinch’s reaction to the manuscript,

How can a proposition be a series of sounds — if it is the kind of thing one knows or desires or fears or …? Couldn’t one say that a particular is the kind of thing one is acquainted with — e.g. — and a universal the kind of thing one understands — and a proposition the kind of thing one knows, fears … etc. Then the *symbol* for a particular is *a* noise and the symbol for a proposition a series of noises … If you are talking about the symbol all the time then it is clear but it is the thing symbolised which is important (I should have thought) to the Analysis of Mind — … All the behaviourist stuff is perplexing to me (1918),

is arguably the most common reaction to sententialist accounts of attitudes and attitude attributions. We will not reconsider all objections here. Suffice it to note that Burge’s claim that sententialism is ‘more of a curiosity than a contender’ (1980, p. 57) as an interpretation is still widespread. If possible, sententialist accounts of attitudes and attitude attributions are widely considered to be even more of an incredible interpretation than the multiple relation theory of judgment.

So, let’s take stock. As we saw at the end of Sect. 2, if one were to think that for ‘the present King of France’ we should follow Russell and at least some of his considerations for his account, one would find oneself in an unstable position, were one not to follow similar considerations while providing an account of phrases which seem to denote propositions. One could then try to straightforwardly extract from Russell a way to reach a more stable position, by endorsing one or the other of his ways of eliminating propositions, even though this might not have been his motivation for either of those views. But Russell’s ways, after *On Denoting*, to eliminate propositions, that is, his multiple relation theory of judgment and his embryonic sententialist account, are indeed usually considered incredible indeed. Suppose that the widespread view that both the multiple relation theory and sententialism are unworkable is indeed correct, maybe because of the objections we just saw, maybe because of some other reasons. What to do? In the rest of this paper, we will then try to address the following question: Is there a way to reach a more stable position that can be extracted, somehow less straightforwardly, from some other of Russell’s claims in *On Denoting*? Stated differently, could somebody unhappy with the ways in which Russell eliminated false objectives still extract from him something leading to a more stable position?

## ‘That’-clauses as quantificational phrases

The claim that definite descriptions are quantificational is, as we saw above, what we now widely consider as Russell’s *intact core insight*. Could one extend this insight to the phrases we use which seem to refer to propositions? And would this allow us to reach a more stable position?

If we follow the Russell of *On Denoting*, both something like ‘The proposition that Cicero is Tully’ and something like ‘heliocentrism’, which looks like a proper name of a proposition, are indeed quantificational. If a proposition about Apollo means what we get by substituting what the classical dictionary tells us is meant by Apollo, one could see ‘heliocentrism’ as meaning something along the lines of ‘the proposition that the Sun is at the centre of the Solar System’. But we do not seem to refer to propositions only, or in fact primarily, via names or definite descriptions. The phrases that typically lead us to introduce propositions are ‘that’-clauses, as they occur in attitude attributions, truth-ascriptions and the like. Would extending Russell’s intact core insight to ‘that’-clauses and then seeing them as quantificational help us reach a more stable position? For reasons that have, at least explicitly, nothing to do with *On Denoting*, some have indeed suggested quantificational accounts of ‘that’-clauses. According to Shier (1996, p. 227), ‘that Cicero is smart’, as it occurs in an attribution of belief to a subject is an existential quantification over what might be called the ‘finer-grained versions’ of the proposition encoded by the embedded sentence ‘Cicero is smart’. The idea is that a belief ascription is used to *characterize*, though not to *specify*, the content of the belief. Bach (2002, p. 227) similarly holds that the ‘that’-clause occurring in a belief attribution does not specify which sort of that-Cicero-is-smart belief the subject has, though he leaves it open how this is to be developed. Also Recanati asks: ‘Can ‘that’-clauses be considered as quantified phrases? Why not?’ (2004, p. 231). He then suggests:

we can treat a ‘that’-clause as, in effect, a restricted existential quantifier, and paraphrase it as ‘For some *p* such that *p* is true iff S’ (where ‘*p*’ now is an objectual variable ranging over truth-evaluable entities, and ‘S’ stands for the sentence embedded in the ‘that’-clause). ‘John believes that grass is green’ is therefore equivalent to

[∃p: TRUE (*p*) iff GREEN (grass)] BELIEVES (John, *p*)

that is, ‘for some *p* such that *p* is true iff grass is green, John believes *p*’ (ibid.).

None of these accounts is developed in detail, but we can still ask whether these accounts could help us reach a more stable position, by positing entities for ‘that’-clauses that would be tolerable for our vivid sense of reality. The invocation of finer-grained versions of propositions and Recanati’s claim that ‘‘*p*’ now is an objectual variable ranging over truth-evaluable entities’, make it clear that, on these accounts, we still have objective falsehoods.

The way in which Russell’s *intact core insight* has been extended to ‘that’-clauses hence does not seem to address, at least not on its own, our worry. But, as we will see in the next section, another of *On Denoting*’s claims might indeed be able to help.

## Imagining and imagining that

In *On Denoting*, Russell claimed:

‘the difference between A and B’ has a denotation when A and B differ, but not otherwise. This difference applies to true and false propositions generally. If ‘*a*R*b*’ stands for ‘*a* has the relation R to *b*,’ then when *a*R*b* is true, there is such an entity as the relation R between *a* and *b*; when *a*R*b* is false, there is no such entity. Thus out of any proposition we can make a denoting phrase, which denotes an entity if the proposition is true, but does not denote an entity if the proposition is false. E.g., it is true (at least we will suppose so) that the earth revolves round the sun, and false that the sun revolves round the earth; hence ‘the revolution of the earth round the sun’ denotes an entity, while ‘the revolution of the sun round the earth’ does not denote an entity. (1905, pp. 490–491).

Could we extract from this of Russell’s claims in *On Denoting* something about ‘that’-clauses? Indeed we can, Russell himself uses them in the passage – it is true that … and false that …. From the passage we can get that out of any sentence we can make a ‘that’-clause, which, whether or not quantificational in character, denotes an entity if the sentence is true, but does not denote an entity if the sentence is false. For example, ‘that the earth revolves round the sun’ would be taken to denote an entity, while ‘that the sun revolves round the earth’ and ‘that the present King of France is bald’ would not denote an entity.

By endorsing this account, we would indeed reach a stable position: exactly as ‘the present King of the United Kingdom’ does denote an entity while ‘the present King of France’ does not, to eliminate shadowy kings, so we would eliminate false objectives. True objectives, on the other hand, the things we do believe when we believe correctly, would then be identical to facts, those things composed of existent objects, properties that are indeed instantiated by those objects, and relating relations that indeed relate those objects.^5^ Similarly for other attitudes.

It is not completely uncommon to hold that some attitudes relate us to facts, in particular knowledge (see Asher, 1993, pp. 27–31, 57 − 9, 171–212; Geach, 1962; Holton, 2017; Hossack, 2007; Merricks, 2009; Moffett, 2003; Parsons, 1993; Ryle, 1929, 1930, pp. 111–114; Vendler, 1972, 1980. Also King, 2007, p. 152 and Wimmer, forthcoming consider and discuss a similar account). But this claim is typically combined with the claims that, first, facts and propositions are entities of different kinds, and that, second, knowledge and belief are not homogeneous, in that in the context of ‘to believe’, ‘that’-clauses denote propositions, while they denote facts in the context of ‘to know’. The view we are considering here rejects these accompanying claims: all attitudes, if they have an object, have a fact as object, which, if one wants, can be taken to be identical to a proposition, but there are no false propositions.

This might look more than just somehow incredible, for at least two related reasons.

First, this is not a topic in *On Denoting*, but elsewhere, and in different periods, Russell himself considered this view (1904, 1907, 1910), and while surprisingly maintaining that ‘common sense seems to prefer’ it (1904, p. 513), he also thought that it was one we could easily make up our mind against. He raised a number of objections. While we do not need to go into all of them here, it is important, for our purposes, to see one. This is arguably the most obvious objection to the view, that is, that we would end up with there being one thing we believe when we believe correctly, while there would be no thing denoted by a ‘that’-clause when there is not the corresponding fact:

Direct inspection seems to leave no room whatever for doubt that, in all presentations and judgments, there is necessarily an object. If I believe that A is the father of B, I believe something; the subsistence of the something, if not directly obvious, seems to follow from the fact that, if it did not subsist, I should be believing nothing, and therefore not believing. (1904, p. 510. See also 1907, p. 46)^6^

The point re-emerged in 1910, when Russell based his introduction of the multiple relation theory on the claim that we cannot have a discrepancy between true and false beliefs:

We must, therefore, abandon the view that judgments consist in a relation to a single object. We cannot maintain this view with regard to true judgments while rejecting it with regard to false ones, for that would make an intrinsic difference between true and false judgments, and enable us (what is obviously impossible) to discover the truth or falsehood of a judgment merely by examining the intrinsic nature of the judgment. Thus we must turn to the theory that *no* judgment consists in a relation to a single object. (1910, pp. 152–153)

Neither points raised in 1910 are without problems, primarily because it is neither clear what an intrinsic difference is, nor is it clear what would the examining of the intrinsic nature of a judgment amount to. Still, we might find ourselves sympathising with Russell’s take that ‘truths are things of the same kind as falsehoods’ (1912, p. 70) and the idea, lurking behind Russell’s explicit points in 1910, and explicit in 1904, that either we always believe some thing, no matter whether we believe truths or falsities, or we never do.

Crucially for our purpose, though, of understanding how we could reach a more stable position than the one constituted by believing Russell’s theory of descriptions so as to rule out Meinongian kings while not being bothered by false objectives, in *On Denoting* itself we can find the resources to weaken our sympathy for that idea that either the object of thought is always there, or never is. For take something like ‘Berty imagines the present King of France’. It seems that, at least on one reading of the sentence, we have an objectual, non-propositional attitude attributed: ‘to imagine’ relates ‘Berty’ and a definite description. Hence, it seems that according to the *intact core insight* in Russell’s theory of descriptions, we would get, simplifying ‘present King of France’, as anyway none of the details here make any difference,

$$\exists\mathrm{x}(\mathrm{PKFx}\:\&\:\forall\mathrm{y}(\mathrm{PKFy}\to\mathrm{x}=\mathrm{y})\:\&\:\mathrm{I}(\mathrm{b},\mathrm{x}))$$

This is false, as it is not the case that there is something which is the present King of France. As Russell said in *On Denoting*, ‘where C stands for any statement about him … every proposition of the form ‘C (the present King of France)’ is false’ (1905, p. 482), hence including the case in which C is the statement that he is imagined by Berty. Invoking secondary occurrences does not seem possible: on an objectual, non-propositional reading of the sentence, there is nothing that could count as creating the secondary occurrence, no operator, no connective and no *so-and-so*, where ‘the so-and-so must be a proposition’ (1905, p. 489). More generally we are not in front of a case in which ‘the present King of France’ occurs ‘in a proposition *p* which is a mere constituent’ (ibid., 489) of the full ‘Berty imagines the present King of France’. The ‘the present King of France’ can only be taken to have primary occurrence and, indeed, ‘all propositions in which ‘the King of France’ has primary occurrence are false’ (ibid., 490). While it might seem that you are imagining the present King of France, there is *no thing* you are imagining, while there would well be *one thing* you were imagining, or thinking about, were you to imagine or think about the present King of the United Kingdom. The details of Russell’s theory of descriptions, or the ways in which it has been amended since 1905, do not matter for our purposes. All that matter is that what is still widely considered as his *intact core insight*, that is, the insight that ‘the present King of France’ is quantificational, together with the prima facie correct claim that, at least on one reading, ‘to imagine’ or ‘to think about’ are relational predicates relating the denotations of the terms flanking the predicate, lead to there being no thing you imagine when you imagine the present King of France, while there is something you imagine when you imagine the present King of the United Kingdom. Of course, ‘to imagine’ is an *intentional transitive*, so there might well be a reading on which what follows it is not to be taken to behave as usual. But none of this is present in *On Denoting*, and at least the following seems to be true: if the present King of France genuinely does not exist, then indeed it is neither bald, not imagined by us, at least on one reading of ‘to imagine’, the one which allows movement and quantifying in. But then if one might find oneself happy to say that there is, at least on one reading of ‘to imagine’, no thing one imagines when the present King of France is imagined, why rejecting flat out the claim that there is something you imagine when you imagine that the earth revolves round the sun, while there is no thing you imagine when you imagine that the sun revolves round the earth? This would be another way to reach an unstable position. To see that the position would indeed be unstable, we can also see that at least some of the moves that can be attempted to make it more palatable to accept that, at least on one reading, there is no thing you imagine when you imagine the present King of France, could be made also concerning ‘that’-clauses, and imagining-that, believing-that and the like. We do not need to go into all these possible moves, considering briefly a couple should suffice.^7^ One could hold that while there is no object one imagines when they seem to imagine the present King of France, or Apollo, if we follow *On Denoting*, this does not mean that there is no act of the imagination having a certain content, it exclusively means that the object of the imagination cannot be the alleged denotation of ‘the present King of France’, as there is not one such denotation. If we dislike senses because of Russell’s rather curious difficulties or because of any other reason, we could take the content to be an image, or a picture (Goodman, 1976). The very same move can indeed be attempted concerning imagining-that and the like. While there might be no fact and then no thing we imagine when we imagine that the sun revolves round the earth, this does not mean that there was no act of imagination and again we can say that we imagined a sentence or a picture. Or we can maintain that while sometimes there is and sometimes there is not the thing we imagined, we are always related to various of the relata Russell himself employed in his multiple relation theory. Maybe Russell himself would have been happy with this combination of claims, and one could consider his multiple relation theory to be a bit less unworkable, if so understood. According to these claims, it would not be the case that *the subsistence of the something follows from the fact that*,* if it did not subsist*,* I should be believing nothing*,* and therefore not believing*. *Believing nothing* would not need to be equated to *not believing*, exactly as *imagining nothing*, which Russell’s theory of descriptions would lead to in the case of imagining Apollo or the present King of France, would not need to be equated to *not imagining*.^8^ Moreover, one could even claim that while one were to believe and imagine no thing, were they to believe that the sun revolves round the earth or to imagine the present King of France, still there would be a way to render ‘S believes something’ and ‘S imagine something’ as true. Even at the peak of his commitment to his multiple relation theory, of course Russell did not abandon the idea that we do speak as if there is an object of thought when we ascribe attitudes (MacBride, 2013), no matter whether true or false. Exactly as we can say that from ‘S imagines the present King of France’, ‘S imagines something’ follows, if we take the quantifier to be a substitutional, non-committal, quantifier, just coming out of the fact that ‘the present King of France’ is a grammatical unit, apt to be substituted for by a quantifier, or ‘it’ or ‘that’, so we can easily obtain ‘S believes something’, if we take the quantifier to be again a non-committal substitutional one, permitted by the fact that ‘that’-clauses are grammatical units. Surely, all these moves just briefly outlined are far from unproblematic, but, and this is all that matters here, they are equally problematic when it comes to imagining the present King of France and imagining that the present King of France is bald.

So, it seems that Russell’s own main point against the view is not insurmountable, and it is moreover a point that can be surmounted by using the very same resources that his theory of description seems to need in order to account for something like ‘Bertie imagines the present King of France’. But there is also another, although related, reason to take the view as incredible. According to the view, something like ‘S believes that the sun revolves round the earth’ is not true. For the ‘that’-clause does not denote anything, given that there is no fact that the sun revolves around the earth. But then the full attribution should be taken to lack a truth-value. But was not there a time where a number of people did indeed believe that the sun revolves around the earth?

Concerning this second reason to be dissatisfied with the view, the first thing to note, given our purposes, is that, as we stated above, according to Russell’s theory of descriptions, there seems to be at least one reading of something like ‘S imagines the present King of France’ on which it has to be false. So both in the case of ‘to imagine’ and in the case of ‘to imagine that’ we seem not to get the truth-value we want. In the case of ‘to imagine’ we seem to get the wrong truth-value, whereas in the case of ‘to imagine that’ we seem not to get one. Yet, in both cases we do not seem able to get the right truth-value. So again, this is not only a problem for the view, but also arises from the theory of descriptions. Thinking that the problem does rule the view out but does not rule the theory of descriptions out would again lead to an unstable position. Second, also here we have that the very same resources, which could be used to diffuse the severity of the problem in the case of ‘to imagine’, could be used concerning ‘to imagine that’. In both cases, one could hold that although the relevant sentences are not true, there are sentences in their vicinity that are indeed true. For example, while it is not true that Bertie imagined the present King of France, it is true that he had an act of imagination involving an image of a certain kind. In the same way, one cold hold that while it is not true that contemporary followers of Ptolemy believe that the sun revolves round the earth, it is true that they would accept the sentence ‘The sun revolves round the earth’ and would take as accurate a picture of the Ptolemaic system. Moreover, to further support their claim, one could even add that our intuitions about the truth-values of sentences such as ‘S imagines the present King of France’ and ‘S believes that the sun revolves round the earth’ are in fact due to not keeping those sentences apart from those other sentences in their vicinity. This would not be an unexplored move when it comes to trying to account for truth-values that might not look correct. In the very context of attitude attributions, for example, some of those Russellians that take propositions to be abstract objects made of things like you, me, mountains and snows, ordered in a particular way, have helped themselves with this very move. According to such a Russellian view of propositions, if Bertie believes that Cicero is an orator, he also believes that Tully is an orator. To explain away the intuition that Bertie can believe the first without believing the second, it has been suggested that we should look at both what sentences such as ‘Bertie believes that Cicero is an orator’ *mean*, and what they *pragmatically convey*. The details do not matter here^9^, but one could for example take the sentence to convey that Bertie would be willing to phrase his own belief using the words ‘Cicero is an orator’, which we can suppose is true. This is not what ‘Bertie believes that Tully is an orator’ conveys, which is instead something along the lines that Bertie would be willing to phrase his own belief using the words ‘Tully is an orator’, something that can well be false in the circumstance under consideration. What is pragmatically conveyed by the two sentences can indeed differ in truth-value and, the explanation continues, this is the reason why we might think that the two sentences themselves can different in truth-value. But this is not genuinely the case. Rather, it is due to the mistake of thinking that what is pragmatically suggested by the two sentences affect their truth-conditions. This move raises a number of questions, and it is not without problems. But this does not matter for our purposes. What matters to us is, first, that there are ways to explain away some intuitions we might have concerning the truth-values of some attitude attributions, and, second, that these ways to explain away some of our intuitions might also be needed to explain away the intuition we might have that it might be true that a subject imagined the present King of France. The view that there is an object we are related to when we believe correctly, which is a fact, but there is no object we are related to when we believe falsely might indeed sound incredible. But not even this second reason to rule the theory out seems able to completely doom it, and rejecting the view because of this second reason, while accepting the core insight in Russell’s theory of descriptions, would again lead to an unstable position.

## Conclusion

Russell concludes on *On Denoting* by admitting that his theory of descriptions does not ‘have such a simplicity as one might have expected’ (1905, p. 493), while begging the reader to be charitable nonetheless. We have been charitable to that view, so much so that what we take to be the core of Russell’s theory of descriptions is now widely believed correct. Among the reasons why this is the case is also the common dislike for Meinongian kings. While it is far from clear that they infringe the law of contradiction, they indeed seem to offend the vivid instinct of many of us as to what is real. But false Meinongian objectives seem on a par, so that rejecting non-existent kings and keeping false-objectives seem an unstable position. Russell himself, for a period after *On Denoting*, eliminated true objectives together with the false ones. We now typically accept both truth and false objectives. But from *On Denoting* we seem able to extract another option: while Russell did not venture into it, we can retain true objectives, while rejecting false ones, exactly as we can keep existing kings while eliminating the non-existent ones. This surely leads to issues, as we would end up believing no thing when we believe falsely. But the view would allow us to finally solve the problem, which troubled Russell and still vexes many of us, of what the relationship between facts and propositions is. According to the account we are suggesting, either propositions do not exist, and the problem is solved, or true propositions, as the things we believe, are indeed identical to facts, while false propositions simply are not, do not exist, and their relationship to facts is then not something we need to establish. For somebody whose sense of reality goes together with a desire for parsimony, rejecting all propositions, or identifying true ones, the only existing ones, with facts, does not simply solve the problem of understanding what the relationship between facts and propositions is, but will have also the welcome result of not having to posit entities of both kinds as distinct^10^. The theory, exactly as with Russell’s theory of descriptions, cannot be taken to have such a simplicity as one might have expected or hoped. For example, it will surely be difficult to explain away the intuition that we always believe some thing, no matter what we believe, and the intuition that some attributions attributing falsities via ‘that’-clauses are true. Moreover, the theory will need resources to overcome Russell’s other objections and surely still others too. But this is true of all theories and, as Russell taught us, it is unclear why it would be acceptable to simply follow the temptation to make one’s mind up against a view just because it looks somehow incredible.

## Footnotes

1 In 1910, Russell claimed: ‘If we allow that all judgments have Objectives, we shall have to allow that there are Objectives which are false. Thus there will be in the world entities, *not dependent upon the existence of judgments*, which can be described as objective falsehoods. This is in itself almost incredible: we feel that there could be no falsehood *if there were no minds to make mistakes*.’ (1910, p. 152, my emphasis). Could the so-called *act theoretic propositions* be able not to offend a vivid sense of reality? According to act-theoretic accounts, entertaining a proposition is the ‘natural cognitive relation’ (Soames, 2010, p. 106) of predicating something of something else (ibid., 81). Out of these particular performances of entertaining (ibid., 106), happening at a particular time and place, we can define propositions. For example, the proposition that Cicero is Roman can be identified with the act/event type of which predicating being Roman of Cicero at a particular time and place is an instance (ibid., 103). So, it might seem that according to the account, false propositions seem *dependent* on the existence of judgment, and can then be tolerated by our vivid sense of reality. But Soames himself maintained that things are not so easy: ‘not all propositional attitudes — e.g. believing and assuming — require occurrent cognitive events, but may sometimes be fully dispositional, agents may sometimes hold propositional attitudes without performing any acts of predication, or experiencing any predicational events’ (2014, p. 229 f.2). More generally, if we want to have all the propositions we need, we need to have some that are linked to no particular judgment, so that, to use Collins’ phrase, we end up with ‘the redundancy of the act’ (2018). Hence, from the point of view of our vivid sense of reality, false act-theoretic propositions are arguably on a par with those made of senses, or Russellian propositions.

2 It is worth stressing here that if one were to think that for Russell his theory of descriptions and his multiple relation theory were intimately connected, then arguably Russell would not have taken the core insight of his theory of descriptions to be that definite descriptions are quantificational, as we currently do, but rather that they are incomplete symbols.

3 It is not clear what the objections are, and neither is it clear how many there are. Similarly, it is not obvious that Russell felt as badly as it might seem from the words in the letter by Wittgenstein and the one by Russell quoted above. For a recent discussion of all this, see Shieh, 2024, and references there.

4 This, it is worth stressing, was explicitly not his view when he was believing in propositions near the time of *On Denoting*. In the *Principles*, he for example claimed: ‘a proposition, unless it happens to be linguistic does not itself contain words: it contains the entities indicated by words’ (1903, p. 48).

5 It is worth remarking that this would arguably resonate well with the pre-*On Denoting* Russell and his playing with the identity theory of truth. On this, see Alford-Duguid & Amijee, 2022, pp. 189–191.

6 As Proops claims, ‘it is clear … that Russell is impressed by the apparent validity of inferences of the form: ‘A believes that the sun orbits the earth; so ∃x [Believes (A, x)].’ … At this stage, however, Russell does not take the argument to be uncontroversially valid. For he sees paradoxes ‘analogous to that of the Liar’ as casting doubt on the idea that belief can be treated as a relation between a believer and a proposition’ (2011, pp. 186–187). For a discussion of the paradox that would be generated by propositions, see Alford-Duguid & Amijee, 2022, pp. 193–194 and references there.

7 For a discussion of other moves, see Sainsbury, 2018, chap. 6 in particular.

8 This move would also help address the first of Russell’s puzzles in *On Denoting* and related issues concerning how fine-grained things are. Of course if ‘that’-clauses denote facts, we might seem to end up with objects that are not fine-grained enough. If we say that attitudes come also with pictures, sentences and other relata, we could still reach something fine-grained enough, even though this would not be the *relatum* in attitude attributions. It is worth stressing that while Russell’s theory of propositions in *On Denoting* does solve the first puzzle about Scott and the author of Waverly, it is far from clear that Russellian propositions solve all similar puzzles. First, as we already saw above, it is unclear that ‘Apollo’ is to be taken as contributing a definite description. Second, it seems that gentlemen in Europe and everybody else can wish to know whether courgettes are zucchini (Crawford, 2004, p. 224; Salmon, 1989/2007, p. 212), so that we seem to have informative identities concerning predicates and properties. It is not clear how these are to be accounted for, no matter whether we claim that ‘that’-clauses denote facts or propositions not made of senses.

9 For the various options and for discussion, see Barwise & Perry, 1983, pp. 258–264; Berg, 1988, p. 363; 2012, pp. 63–70; McKay, 1981; Peacocke, 1975, pp. 127–128; Richard, 1987; Salmon, 1986﻿, pp. 58–60; 114–118; 1989/2007, pp. 198–199; Saul, 1997, p. 106, Soames, 1987, 2002, pp. 228–229; Urmson, 1968, pp. 116–118.

10 This might arguably be traced back to Russell himself, around the time of *On Denoting*. For, as Alford-Duguid and Amijee claim, ‘Russell [(1904)] … may also appear to invite an argument against correspondence from parsimony: Russell may have found it distasteful to countenance both true propositions and facts, as one might find countenancing pervasive, undetectably coincident dopplegängers distasteful. That is, of course, rather speculative’ (2022, p. 203 f.14).
